# Prognosis of pathogen-proven acute respiratory distress syndrome diagnosed from a protocol that includes bronchoalveolar lavage: a retrospective observational study

**DOI:** 10.1186/s40560-020-00469-w

**Published:** 2020-07-23

**Authors:** Michihito Kyo, Koji Hosokawa, Shinichiro Ohshimo, Yoshiko Kida, Yuko Tanabe, Nobuaki Shime

**Affiliations:** Department of Emergency and Critical Care Medicine, Graduate School of Biomedical and Health Sciences, Hiroshima University, 1-2-3 Kasumi, Minami-ku, Hiroshima, 734-8551 Japan

**Keywords:** Pneumonia, BAL, Mimicker, Common risk factor, ARDS, Sepsis, ICU

## Abstract

**Background:**

To treat patients with acute respiratory distress syndrome (ARDS), it is important to diagnose specific lung diseases and identify common risk factors. Our facility focuses on using bronchoalveolar lavage (BAL) to identify precise risk factors and determine the causative pathogen of ARDS within 24 h of intensive care unit (ICU) admission. This study evaluated the prognoses of pathogen-proven ARDS patients who were diagnosed or identified with risk factors using a diagnostic protocol, which included BAL, compared with the prognoses of pathogen-unproven ARDS patients.

**Methods:**

This retrospective observational study was conducted in the ICU at a tertiary hospital from October 2015 to January 2019. We enrolled patients with respiratory distress who were on mechanical ventilation for more than 24 h in the ICU and who were subjected to our diagnostic protocol. We compared the disease characteristics and mortality rates between pathogen-proven and pathogen-unproven ARDS patients.

**Results:**

Seventy ARDS patients were included, of whom, 50 (71%) had pathogen-proven ARDS as per our protocol. Mortality rates in both the ICU and the hospital were significantly lower among pathogen-proven ARDS patients than among pathogen-unproven ARDS patients (10% vs. 50%, *p* = 0.0006; 18% vs. 55%, *p* = 0.0038, respectively). Pathogen-proven ARDS patients were independently associated with hospital survival (adjusted hazard ratio, 0.238; 95% confidence interval, 0.096–0.587; *p* = 0.0021).

**Conclusions:**

Our diagnostic protocol, which included early initiation of BAL, enabled diagnosing pathogen-proven ARDS in 71% of ARDS patients. These patients were significantly associated with higher hospital survival rates. The diagnostic accuracy of our diagnostic protocol, which includes BAL, remains unclear.

## Background

Acute respiratory distress syndrome (ARDS) is a life-threatening disease with a mortality rate of ~ 40% [1]. The Berlin definition defines ARDS as respiratory distress occurring within 7 days of recognizing a common risk factor [2]. However, some patients are diagnosed with ARDS based on pathophysiological parameters but without a proven etiology or causative pathogen [3]. Thus, studies examining ARDS often include heterogeneous syndromes as well as ARDS mimickers [4].

The bronchoalveolar lavage (BAL) examination is used to differentially diagnose respiratory diseases, including ARDS. One study found that of ARDS patients who underwent BAL, 56% presented microbial pathogens and were definitively diagnosed with pneumonia, the leading risk factor for ARDS [5]. Therefore, BAL enables performing successful definitive therapy and reduces mortality from ARDS. Gibelin et al. reported that ARDS patients without common risk factors were diagnosed with autoimmune and malignant diseases via BAL examination, and these patients were associated with higher mortality risks [6]. BAL was recently recommended as a method for identifying the ARDS etiology and distinguishing interstitial pneumonia from ARDS [3, 7, 8]. However, the secondary analysis of the LUNG SAFE study revealed that only 9% of ARDS patients underwent BAL [9].

Our facility focuses on diagnosing lung diseases, differentiating interstitial pneumonia, and identifying the ARDS etiology using BAL. Furthermore, we started a protocol for diagnosing or identifying ARDS etiologies via sputum culture, gene analysis, serum testing, BAL analysis, and computed tomography (CT) scans in 2015. This study compared the prognoses of ARDS patients with or without proven causative pathogen using our diagnostic protocol, which includes BAL.

## Methods

### Study design and population

This observational study was conducted in the emergency and medical intensive care units (ICUs) of Hiroshima University Hospital from October 2015 to January 2019. The Institutional Review Board of Hiroshima University approved the study protocol (trial registration: E-1751, registered on 17 September 2019).

We retrospectively reviewed the medical records of ICU patients with respiratory failure at admission and included consecutive patients (aged ≥ 18 years) who were considered to have ARDS from pathophysiological parameters and stayed in the ICU for more than 24 h. Clinically defined ARDS was diagnosed and categorized as mild, moderate, or severe according to the Berlin definition. Patients with respiratory distress of unknown etiology were included. Patients who were postoperative or non-medical (including trauma and burns) admission, had interstitial pneumonia, or had do-not-resuscitate orders were excluded.

### Diagnostic protocol

All ARDS patients underwent chest X-rays and CT scans at the timing of diagnosing ARDS if their condition allowed it. After patients were intubated, BAL was performed to determine the ARDS etiologies and causative pathogens. For the BAL procedure, 100–150 mL of normal saline was injected into the wedged bronchi, where a lobar infiltrate was observed on chest CT scans, and gently suctioned. The BAL fluid (BALF) was rapidly Gram-stained, cultured, and underwent cytological analysis on a weekday. When Gram staining of the BALF revealed no microorganisms, the BALF was analyzed via polymerase chain reaction (PCR) for *Mycobacterium* spp. and *Mycoplasma pneumoniae*, and Loop-mediated isothermal amplification (LAMP) for *Legionella pneumophila*. Urinary antigen testing was also performed for *Streptococcus pneumonia* and *Legionella pneumophila*. For immunosuppressed patients, we measured the serum beta-D-glucan, analyzed the BALF for the *Aspergillus* antigen, and performed PCR for *Pneumocystis carinii* and cytology and C7-HRP to detect *Cytomegalovirus* spp. During the regional epidemic season, reverse-transcription PCR was performed on the BALF to test for the *influenza virus*. When causative pathogens were not identified or the precise cause of ARDS could not be determined, we further analyzed the BAL cell differentials to determine the etiology of ARDS, and this sometimes revealed evidence of interstitial pneumonia. However, potential pathogens were only identified during the initial analysis of the BAL fluid in the present study.

Immunological testing, including laboratory tests for proteinase-3-anti-neutrophil cytoplasmic antibodies (ANCA), myeloperoxidase-ANCA, anti-basement membrane antibody, and antinuclear antibody, were also performed.

### Definition

ARDS etiology was determined via a diagnostic protocol, which included BAL. Pathogen-proven ARDS was defined according to the following risk factors: (1) pneumonia with an identified causative pathogen, (2) nonpulmonary sepsis with an identified causative pathogen, and (3) aspiration pneumonia. Pneumonia was diagnosed from at least one of the following: body temperature > 38.0 °C; white blood cell count > 12,000/mm^3^ or < 4000/mm^3^; altered mental status; and a positive microbial culture including bacteria, fungi, and/or a virus [10], in addition to new regional or lobar infiltration on chest radiographs and CT scans. Nonpulmonary sepsis was diagnosed as an increased Sequential Organ Failure Assessment (SOFA) score of ≥ 2 points and identification of an infectious source other than the lungs. Aspiration pneumonia was diagnosed on the basis of a characteristic clinical history (witnessed aspiration), the presence of risk factors (lower level of consciousness, an impaired cough reflux or impaired swallowing), and radiographic findings, including the presence of infiltrates in gravity-dependent lung segments [11].

### Management

The ventilator management was lung protective ventilation. Patients with partial pressure of arterial oxygen (PaO_2_)/fraction of inspiratory oxygen (F_I_O_2_) ratio < 100 were considered using neuromuscular blockage, initiating prone positioning and veno-venous extracorporeal membrane oxygenation (ECMO) which were performed for some but not all patients. Veno-venous ECMO was initiated according to the findings of the CESAR trial [12], i.e., when the Murray score (derived from all four variables: PaO_2_/FIO_2_ ratio, positive end-expiratory pressure, lung compliance, and chest radiographic appearance; and when FIO_2_ = 1) was ≥ 3.0 or the pH was < 7.20, or the patient did not respond to protective lung ventilation and prone positioning (SaO_2_ < 90% or pH < 7.20).

### Data collection

We collected demographic data, including age, sex, past illness history, SOFA score, Acute Physiology and Chronic Health Evaluation (APACHE) II score, and ARDS severity upon ICU admission. We also recorded the lowest PaO_2_/F_I_O_2_ ratio, tidal volume, ventilator parameters, and ARDS therapy used (e.g., neuromuscular-blocking agents, corticosteroid therapy, initiation of prone positioning, hemodialysis, ECMO, and tracheostomy). The clinical outcomes were ventilator management duration, length of the hospital and ICU stays, and mortality.

### Statistical analysis

Values are presented as medians (interquartile range; IQR) or numbers (percentage) as appropriate. Categorical variables were compared between pathogen-proven and pathogen-unproven ARDS patients using Fisher’s exact tests. Continuous variables were compared using Mann-Whitney *U* tests. Cox regression analysis was performed to assess the pathogen-proven ARDS relative to hospital mortality, and the results are shown as hazard ratios. Factors with *p* value < 0.05 in the univariate analyses and pathogen-proven ARDS were entered into the multivariate model. All statistical analyses were conducted using the JMP statistical software (version 14.0.0; SAS, Cary, NC, USA).

## Results

### Prevalence of pathogen-proven ARDS

**Fig. 1:**
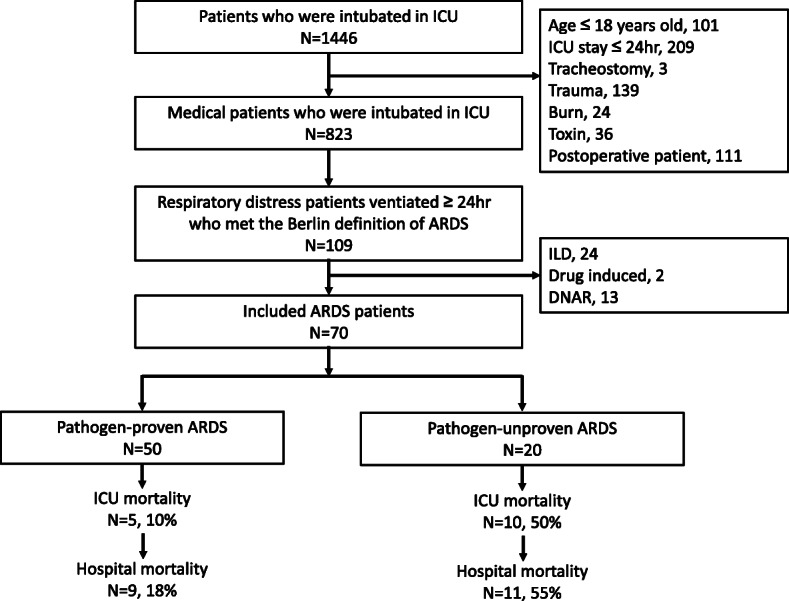
Flowchart of enrolled patients. DNAR, do not attempt resuscitation; ICU, intensive care unit; ARDS, acute respiratory distress syndrome; ILD, interstitial lung disease

In total, 1446 patients were intubated, of which, 109 met the Berlin definition of ARDS and stayed in the ICU for more than 24 h. Finally, 70 ARDS patients who met the inclusion criteria were analyzed (Fig. 1). Fifty patients (71%) had pathogen-proven ARDS as per the diagnostic protocol that included BAL.

### ARDS patient characteristics

Table 1 shows the patients’ baseline characteristics. The median age was 66 years (range, 57–74 years), and 42 patients (61%) were men. The median SOFA score was 11 (9–13); the median APACHE II score was 28 (24–32). In this cohort, age, SOFA score, APACHE II score, ARDS severity, and mechanical ventilation parameters did not significantly differ between pathogen-proven and pathogen-unproven ARDS patients.

**Table 1:** Patient characteristics and ventilator parameters on the day of admission

	All patients (***n*** = 70)	Pathogen-proven ARDS group (***n*** = 50)	Pathogen-unproven ARDS group (***n*** = 20)	***p***
Age, year	66 (57–73)	67 (59–74)	60 (43–71)	0.097
Male	43 (61)	32 (64)	11 (55)	0.589
SOFA score	11 (9–13)	11 (9–13)	11 (10–13)	0.700
APACHE II score	29 (24–32)	29 (24–32)	28 (25–31)	0.745
Transferred from other hospital	26 (37)	21 (42)	5 (25)	0.274
Prior use of antibiotics	29 (41)	18 (36)	11 (55)	0.183
Comorbidities				
Heart failure	1 (1)	0 (0)	1 (5)	0.286
Stroke	6 (9)	5 (10)	1 (5)	0.666
COPD	7 (10)	4 (8)	3 (15)	0.399
Renal failure	6 (9)	3 (6)	3 (15)	0.343
Malignancy	16 (23)	13 (26)	3 (15)	0.529
Liver failure	19 (27)	14 (28)	5 (25)	1.000
Immunosuppression	17 (24)	11 (22)	6 (30)	0.543
Severity of ARDS (Berlin definition)				0.620
Mild	10 (14)	6 (12)	4 (20)	
Moderate	35 (47)	27 (50)	8 (40)	
Severe	27 (39)	19 (38)	8 (40)	
Mechanical ventilation				
PaO_2_/F_I_O_2_	127 (85–179)	127 (82–176)	135 (96–196)	0.413
F_I_O_2_	0.60 (0.40–0.76)	0.60 (0.45–0.80)	0.53 (0.40–0.74)	0.377
PEEP	11 (8–14)	10 (8–14)	12 (10–14)	0.155
Driving pressure	13 (10–16)	12 (10–14)	14 (10–16)	0.483
TV	455 (389–529)	460 (397–524)	455 (364–550)	0.716
TV/PBW	8.0 (7.0–9.4)	8.0 (7.0–10.0)	8.0 (7.1–9.2)	0.721
Septic shock	26 (37)	24 (48)	2 (10)	0.003

Values are given as the median (interquartile range) or number (%). *p* values were calculated via Fisher’s exact test or the Mann-Whitney *U* test

*ARDS* Acute respiratory distress syndrome, *SOFA* Sequential Organ Failure Assessment, *APACHE* Acute Physiology and Chronic Health Evaluation, *COPD* chronic obstructive pulmonary disease, *PaO*_*2*_ partial pressure of arterial oxygen, *F*_*I*_*O*_*2*_ fraction of inspiratory oxygen, *PEEP* positive end-expiratory pressure, *TV* tidal volume, *PBW* predicted body weight

### ARDS etiology

In the 50 pathogen-proven ARDS patients, pneumonia was the most common risk factor (*n* = 31), followed by sepsis (*n* = 13), and aspiration (*n* = 6; Table 2). Of the 31 pneumonia patients, 20 had bacteria, 4 had viruses, 4 had fungi, and 3 had both viruses and fungi in their BAL. *Streptococcus pneumonia* was predominant (*n* = 7) among the bacterial pneumonia patients. The *influenza* virus was predominant (*n* = 6) among viral pneumonia patients.

**Table 2:** Causative microorganisms of acute respiratory distress syndrome

*N* = 50		
Pneumonia (*N* = 31)		
Bacteria (*N* = 20)	*Streptococcus pneumonia*	7
	MRSA	2
	*Legionella pneumophila*	4
	MSSA	2
	*Klebsiella pneumonia*	1
	*Schewanella algae*	1
	*Moraxella catarrhalis*	1
	*Enterobacter aerogenes*	1
	*Haemophilus influenza*	1
Virus (*N* = 7)	*Influenza* virus	6
	*Cytomegalovirus*	1
Fungi (*N* = 7)	*Aspergillus* spp.	3
	*Pneumocystis jirovecii*	3
	*Cryptococcus neoformans*	1
Aspiration (*N* = 6)		
Sepsis (*N* = 13)		
	*Streptococcus pyogenes*	4
	*Escherichia coli*	3
	MRSA	2
	*Peptostreptococcus* spp., *prevotella oralis*	1
	*Morganella morganii*	1
	*Klebsiella pneumonia*	1
	*Klebsiella oxytoca*	1
	*Leptotrichia trevisanii*	1

Aspergillus spp. in pneumonia patients and *Escherichia coli* in sepsis patients were duplicated. Of the 31 patients with pneumonia, three had both viruses and fungi as causative pathogens

*MRSA* methicillin-resistant *Staphylococcus aureus*, *MSSA* methicillin-sensitive *Staphylococcus aureus*

### Treatment and outcomes of the ARDS patients

The treatment options used (e.g., neuromuscular-blocking agents, prone positioning, corticosteroid therapy, and veno-venous ECMO initiation) did not significantly differ between the groups (Table 3). The ICU-free days during a 28-day period in the pathogen-proven ARDS group was significantly longer than in the pathogen-unproven ARDS group (13 [5–16] vs. 1 [0–15], *p* = 0.034). The overall ICU mortality rate was 21%; the hospital mortality rate was 29%. The ICU and hospital mortality rates were significantly lower in ARDS patients with identified etiologies (10% vs. 50%, *p* = 0.0006; 18% vs. 55%, *p* = 0.0038, respectively).

**Table 3:** Therapy and outcome

	All patients (***N*** = 70)	Pathogen-proven ARDS group (***N*** = 50)	Pathogen-unproven ARDS group (***N*** = 20)	***p***
Therapy				
Neuromuscular blocking agents	12 (17)	8 (16)	4 (20)	0.732
Corticosteroid therapy	29 (41)	20 (40)	9 (45)	0.791
Prone position	5 (7)	3 (6)	2 (10)	0.619
Hemodialysis	21 (30)	15 (30)	6 (30)	1.000
VA ECMO	5 (7)	4 (8)	1 (5)	1.000
VV ECMO	12 (17)	11 (22)	1 (5)	0.158
Tracheostomy	28 (40)	20 (40)	8 (40)	1.000
Appropriate antibiotic therapy for causative pathogens within day 3	–	48 (96)	–	–
Outcome				
Ventilator-free days of 28 days	16 (0–20)	18 (7–20)	4 (0–22)	0.112
ICU-free days of 28 days	13 (0–16)	13 (5–16)	1 (0–15)	0.034
ICU mortality	15 (21)	5 (10)	10 (50)	0.0006
Hospital-free days of 28 days	0 (0–6)	0 (0–6)	0 (0–7)	0.613
Hospital mortality	20 (29)	9 (18)	11 (55)	0.0038

Values are given as the median (interquartile range) or number (%). *p* values were calculated using Fisher’s exact test or the Mann-Whitney *U* test

*ARDS* acute respiratory distress syndrome, *VA ECMO* veno-arterial extracorporeal membrane oxygenation, *VV* veno-venous, *ICU* intensive care unit

### Factors associated with hospital mortality

Univariate analyses showed that pathogen-proven ARDS (hazard ratio [HR], 0.265; 95% confidence interval [CI], 0.109–0.647; *p* = 0.004) and higher SOFA scores (HR, 1.211; 95% CI, 1.068–1.374; *p* = 0.0028) were significantly associated factors with hospital mortality (Table 4). Pathogen-proven ARDS was significantly associated with hospital mortality after adjusting for SOFA score (HR, 0.238; 95% CI, 0.096–0.587; *p* = 0.0021).

**Table 4:** Univariate and multivariate analyses of factors associated with hospital survival

Variables	Univariable HR	95% CI	***p***	Multivariable HR	95% CI	***p***
Pathogen-proven ARDS	0.265	0.109–0.647	0.004	0.238	0.096–0.587	0.0021
Age (per year decrease)	0.974	0.942–1.008	0.126			
Male	0.751	0.302–1.869	0.542			
SOFA score (per 1 increase)	1.211	1.068–1.374	0.0028	1.226	1.082–1.390	0.0015
APACHEIIscore (per 1 increase)	1.030	0.966–1.101	0.363			
PaO_2_/F_I_O_2_	1.014	0.940–1.090	0.715			
COPD	1.941	0.560–6.730	0.332			
Liver failure	1.869	0.762–4.586	0.184			
Corticosteroids	1.164	0.478–2.830	0.739			
Hemodialysis	2.356	0.956–5.806	0.069			
VV ECMO	0.834	0.243–2.867	0.769			

*HR* hazard ratio, *CI* confidence interval, *ARDS* acute respiratory distress syndrome, *SOFA* Sequential Organ Failure Assessment, *APACHE* Acute Physiology and Chronic Health Evaluation, *COPD* chronic obstructive pulmonary distress, *VV ECMO* veno-venous extracorporeal membrane oxygenation

## Discussion

In the present study, 71% of ARDS patients had pathogen-proven ARDS. To our knowledge, this was the first study to investigate the prognostic impact of a diagnostic protocol that included BAL in ARDS patients. The hospital mortality rate of pathogen-proven ARDS patients was lower than that of pathogen-unproven patients after adjusting for SOFA scores.

A nationwide survey in Japan revealed that 34% of ARDS patients had pneumonia, and all ARDS patients had risk factors [13]. Conversely, a survey conducted in the USA from 2006 to 2014 revealed that approximately 45% of ARDS patients had pneumonia, and 16% had no specific risk factors [14]. The discrepancy between these findings may have occurred because of the ambiguous diagnosis of ARDS risk factors, which depends on BAL for detecting microorganisms that cause pneumonia or the vague clinical criteria for pneumonia. In our setting, BAL-based detection systems, especially LAMP for *Legionella pneumophila* and PCR for *Pneumocystis jirovecii*, *influenza viruses,* and *cytomegaloviruses,* contributed to detecting many causative organisms. This is consistent with the findings of previous studies and supports aggressively using BAL to increase the ability to diagnose pneumonia as an ARDS etiology [15–17].

The reduced mortality of pathogen-proven ARDS patients in this study may be explained as follows. First, ARDS patients with no common risk factors included those with autoimmune and idiopathic diseases, and the absence of common risk factors has been associated with increased mortality in ICUs [6, 18]. Second, the outcomes (i.e., development of acute lung injury/ARDS or mortality) of patients with infections can be improved via early and appropriate antimicrobial therapy [19–21]. In addition, precise detection of microorganisms shortens the duration of empiric antibiotic therapy [22], resulting in fewer adverse events. Given the overall low performance of BAL (9.4%) in a large-scale epidemiological study (LUNG SAFE study) [9], BAL-based diagnostic approaches should be more widely applied for ARDS patients to help improve their outcomes.

This study had several limitations. First, it was a single-center, retrospective observational study of relatively few patients. In addition, the etiology of the pathogen-unproven ARDS was not determined (Supplementary Table 1). We excluded potential participants with several major ARDS etiologies (e.g., burn, trauma, and drug-induced) and other etiologies (e.g., interstitial pneumonia). Analysis of the BAL fluid revealed no significant pathogens in the pathogen-unproven ARDS patients, and they also did not have non-septic shock or other significant risk factors, such as transfusion or pancreatitis. However, this group could have included “ARDS mimickers” as defined in a previous study [3] and hematological malignancy-related ARDS. The survival rate of ARDS mimickers and hematological malignancy-related ARDS is poor [23], which may explain the poor outcomes in the pathogen-unproven ARDS cohort in the present study, even though there were fewer patients with septic shock in this group. Further studies are required to investigate the clinical characteristics of these subtypes of ARDS. Second, our hospital is a tertiary hospital, and 37% of our patients were transported from other hospitals after antibiotic administration, which may differ among settings. Third, the selection of wedged bronchi for BAL might have affected the sensitivity of pathogen detection. Fourth, regarding viruses, we only tested for cytomegaloviruses and influenza viruses. Therefore, presence of other causative viruses, such as rhinoviruses, adenoviruses, and herpesviruses, is unknown. Applications of currently available, easy-to-use, comprehensive, molecular-based diagnostic systems, such as Fillmarray^TM^, would help increase pathogen detection rates and enable faster treatment, especially for viruses [24]. In addition, we included patients who were on mechanical ventilation for more than 24 h; thus, some severely ill patients may have been excluded, affecting the mortality analysis. Finally, the definition of “pathogen-unproven ARDS” has not been standardized and may include ARDS “mimickers” [3]. However, the definition of ARDS “mimickers” has also not been standardized. These two terms should be precisely defined to accurately categorize the heterogeneity of ARDS.

## Conclusion

Pathogen-proven ARDS patients who were diagnosed via diagnostic work-up that included BAL had lower mortality rates than did pathogen-unproven ARDS patients. Pathogen-unproven ARDS was significantly associated with hospital mortality. The diagnostic accuracy and significance for treatment of the diagnostic protocol, including BAL, should be determined in further studies.

## Supplementary information

**Additional file 1: Table S1.** Etiologies of pathogen-unproven ARDS (*n*=20).

## Data Availability

The datasets used and/or analyzed during the current study are available from the corresponding author on reasonable request.

## Abbreviations

ANCA: Anti-neutrophil cytoplasmic antibodies
APACHE: Acute Physiology and Chronic Health Evaluation
ARDS: Acute respiratory distress syndrome
BAL: Bronchoalveolar lavage
BALF: Bronchoalveolar lavage fluid
CI: Confidence interval
CT: Computed tomography
ECMO: Extracorporeal membrane oxygenation
F_I_O_2_: Fraction of inspiratory oxygen
HR: Hazard ratio
LAMP: Loop-mediated isothermal *amplification*
PaO_2_: Partial pressure of arterial oxygen
PCR: Polymerase chain reaction
SOFA: Sequential Organ Failure Assessment
