# Supplementary material for: Prognosis of pathogen-proven acute respiratory distress syndrome diagnosed from a protocol that includes bronchoalveolar lavage: a retrospective observational study
Source: J Intensive Care. 2020 Jul 23;8:54. doi: 10.1186/s40560-020-00469-w (PMC7376525; doi:10.1186/s40560-020-00469-w)
Supplement: Supplementary file 1 — Additional file 1: Table S1. Etiologies of pathogen-unproven ARDS (n=20). [file 40560_2020_469_MOESM1_ESM.docx]

Table S1. Etiologies of pathogen-unproven ARDS (n=20)

| Etiologies | | n |
| --- | --- | --- |
| Infection-associated ARDS | | 12 |
|  | Suspected pneumonia | 9 |
|  | Suspected non-pulmonary sepsis | 3 |
| Malignant ARDS | | 3 |
|  | Suspected malignant hematological diseases | 2 |
|  | Suspected idiopathic pneumonia syndrome | 1 |
| Unknown etiology | | 3 |
| Coagulopathy-associated ARDS | | 2 |

ARDS, acute respiratory distress syndrome
